# Microstructure and Mechanical Properties of Hypereutectic Al-High Si Alloys up to 70 wt.% Si-Content Produced from Pre-Alloyed and Blended Powder via Laser Powder Bed Fusion

**DOI:** 10.3390/ma16020657

**Published:** 2023-01-10

**Authors:** Jan Henning Risse, Matthias Trempa, Florian Huber, Heinz Werner Höppel, Dominic Bartels, Michael Schmidt, Christian Reimann, Jochen Friedrich

**Affiliations:** 1 Fraunhofer IISB, Schottkystrasse 10, 91058 Erlangen, Germany; 2 Department of Mechanical Engineering, Institute of Photonic Technologies, Friedrich-Alexander-University of Erlangen-Nuremberg, Konrad-Zuse-Straße 3/5, 91052 Erlangen, Germany; 3 Department Material Science and Engineering, Institute I: General Materials Properties, Friedrich-Alexander-University of Erlangen-Nuremberg, Martensstr. 5, 91058 Erlangen, Germany

**Keywords:** additive manufacturing, laser powder bed fusion, hypereutectic Al-high Si alloys, in-situ alloying, microstructure, mechanical properties

## Abstract

Hypereutectic Al-high Si alloys are of immense interest for applications in the automotive, space or electronic industries, due their low weight, low thermal expansion, and excellent mechanical and tribological properties. Additionally, their production by laser powder bed fusion (LPBF) technology provides high flexibility in geometrical design and alloy composition. Since, most of the alloy properties could be improved by increasing the Si content, there is much interest in discovering the maximum that could be realized in LBPF Al-high Si alloys, without the appearance of any material failure. For this reason, in this work the production of Al-high Si alloys with extremely high silicon content of up to 70 wt.% was fundamentally investigated with respect to microstructure and mechanical properties. Highly dense (99.3%) and crack-free AlSi50 samples (5 × 5 × 5 mm^3^), with excellent hardness (225 HV5) and compressive strength (742 MPa), were successfully produced. Further, for the first time, AlSi70 LBPF samples of high density (98.8%) without cracks were demonstrated, using moderate scanning velocities. Simultaneously, the hardness and the compressive strength in the AlSi70 alloys were significantly improved to 350 HV5 and 935 MPa, as a result of the formation of a continuous Si network in the microstructure of the alloy. With respect to the powder source, it was found that the application of powder blends resulted in similar alloy properties as if pre-alloyed powders were used, enabling higher flexibility in prospective application-oriented alloy development.

## 1. Introduction

Hypereutectic Al-high Si alloys, especially with Si content > 30 wt.%, are characterized by preeminent properties, like low density, low thermal expansion, high hardness and stiffness, and excellent wear resistance, which enable numerous applications in the automotive, space and electronics industries [1,2]. All the alloy properties mentioned improve with increasing Si content, hence, there is high industrial interest regarding the commercial availability of AlSi-alloys with Si-content ≥ 50 wt.%. However, during the conventional casting process of such Al-high Si alloys, a coarse and highly brittle primary Si phase is formed, leading to crack formation in the casted parts and reducing their performance [3,4]. The Fabrication via rapid solidification technologies, like spray deposition or cold/hot pressing, can prevent the extensive coarsening of this Si phase and enable the production of alloys up to 90 wt.% silicon [5,6,7]. However, these production methods are limited to rather simple geometries and often require some post-treatment relating to final densification or geometrical finishing.

A promising fabrication technique, which mostly overcomes these limitations, is additive manufacturing (AM), especially the powder bed fusion of metal with laser beam (PBF–LB/M or LPBF) technology, in which complex and dense components are built up via printing layer by layer [8,9]. This technique has already been used to principally investigate Al-high Si alloys with a silicon content up to 50 wt.% (see e.g., [4,10]). A comparison to casted AlSi50 samples, conducted by Jia et al. [4], showed a refinement of the primary Si, from platelike particles, with sizes >100 µm, to polygonal-shaped particles, with sizes <6 µm, using PBF–LB/M, which led to an increase of the compressive strength by almost 50%, to over 650 MPa. Kang et al. [10] demonstrated AlSi50 samples with a maximal hardness of 188 HV0.3, which is approximately 50% higher in comparison to the well-established AM-alloy AlSi10Mg [11]. Despite these promising singular results, knowledge about the microstructure and its correlation to the mechanical properties of these Al-high Si AM-alloys is still limited. So, the findings in the literature about processibility via PBF–LB/M, and the material properties, are not consistent. On the one hand, several researchers reported cracks in AlSi40 [12,13,14] and AlSi50 samples [15]. On the other hand, other findings did not mention any crack formation [4,10,16]. Consequently, further research is needed to clarify the cracking issue. Additionally, the reported density values of AlSi50 samples are widely scattered, from ~98% [10] to ~99.9% [15], so there is a need to clarify if this difference is related to intrinsic material properties, processing or accuracy in characterization. Further, the production of Al-high Si alloys with Si contents higher than 50 wt.% have not yet been demonstrated, which might be correlated with the cracking issue mentioned, as well as with a lack of standardized AM powder sources. 

Therefore, the aims of this work were the following: (1) to extend knowledge about the microstructure and its correlation to the mechanical properties in AM Al-high Si alloys and (2) to prove the feasibility of AM Al-high Si alloys with even higher Si content. A detailed investigation into the correlation of process parameters, especially scan velocity, microstructure, and crack formation, as well as mechanical properties in PBF–LB/M AlSi50 alloys was carried out, followed by extension to an even higher Si content of 70 wt.%. Parallel to these activities, due to the lack of pre-alloyed AlSi70 powder, the effect of the use of powder blends, which were already successfully tested for AlSi40 [13] and AlSi50 [10,15] alloys, was investigated.

## 2. Materials and Methods

### 2.1. Materials

**Figure 1:**
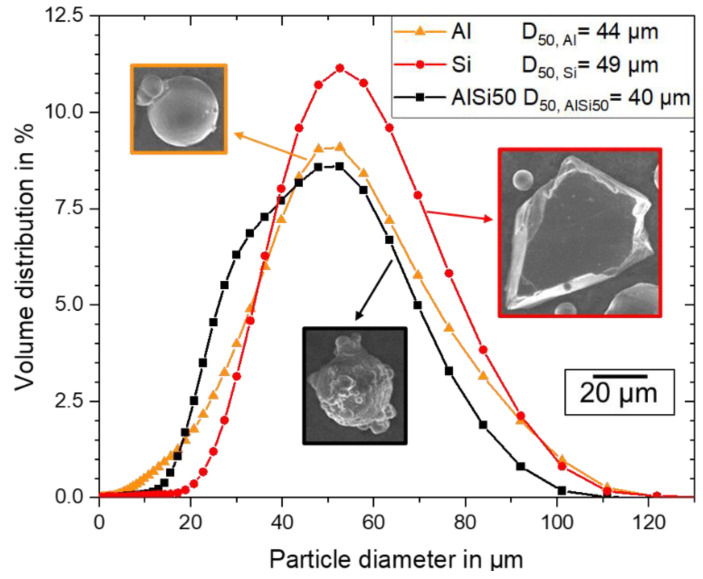
Particle size distribution and SEM images of the elemental Al and Si powders and the pre-alloyed AlSi50 powder measured via laser diffraction.

To investigate the effect of the powder sources on the resulting alloy properties, three different powder types, namely, a pre-alloyed AlSi50 powder, an Al powder, and a Si powder, were used. The particle size distributions, measured by laser diffraction (particle analyzer Beckman-Coulter LS13320) according to ISO 13320-1, and SEM-images from single powder particles are shown in Figure 1. The pre-alloyed AlSi50 powder (gas atomized by NANOVAL GmbH & Co. KG, Berlin, Germany) had a mainly spherical shape with some satellites on the particle surfaces and a skewed particle size distribution, with a medium diameter (D50-value) of 40 µm. The spherical Al99.7 powder (gas atomized by TLS Technik GmbH & Co. Spezialpulver KG, Niedernberg, Germany) and the edged Si powder (Silgrain^®^ from Elkem ASA, Oslo, Norway) had Gaussian particle size distributions with D50-values of 44 µm and 49 µm, respectively.

On the one hand, the pre-alloyed AlSi50 powder (p.) was compared to the more flexible and cheaper approach of using powder blends made of elementary that were only partially gas-atomized (Al + Si). Furthermore, it was also used in blends with silicon (p. + Si) to achieve a higher silicon content of 70 wt.% and to evaluate the different powder blends in its performances. All powders/powder blends were dried at 110 °C for 15 h under vacuum atmosphere and the powder blends were mixed for 1 h in a tumbling mixer after drying before being further processed. 

### 2.2. Sample Fabrication via PBF–LB/M, Preparation, and Characterization

The samples were produced using a mini PBF–LB/M machine from Aconity GmbH, equipped with a single mode fiber laser with up to 1 kW power and a wavelength of 1080 nm. During the fabrication process the building chamber was ventilated with argon gas to keeping the residual oxygen content below 100 ppm. The build space was downsized to a 55 mm diameter platform made of a sandblasted aluminum base plate, with a thickness of 5 mm. 

Based on a preceding process parameter optimization, the laser power, layer thickness, hatch distance, and laser spot size were fixed to 350 W, 50 µm, 120 µm and 72 µm, respectively. Only the influence of the scan velocity was investigated and is discussed in the present article. The laser beam scanned the layer’s geometry in a meandering pattern, which was rotated by 67° in the subsequent layer. The microstructure was examined in 5 × 5 × 5 mm^3^ cubes, which were ground and polished up to a final polishing step with 0.25 µm fumed silica. To determine the porosity, light microscope images of longitudinal cuts were analyzed using a MATLAB script, which converted the image into a binary image and calculated the porosity, as well as the size, of the single pores. With this measurement procedure the smallest resolvable pore diameter was approximately 1 µm, which is significantly smaller than the 20–30 µm which is typically reached by computer topography (CT). The longitudinal cuts of almost all samples built with scan velocities of 1000, 1400, 1600, 2000 and 2400 mm/s (the last only with AlSi50 p.) were inspected via SEM (JEOL 6510, equipped with a secondary electron detector) to investigate the features of the microstructure. Therefore, the cuts were etched with deionized water and HF and metallized with an approximately 3 nm platinum layer. For each sample, nine micrographs were taken and analyzed via ImageJ regarding the primary Si particle sizes. The fractions of the fine and coarse areas were determined, based on light microscopic images. 

The hardness was measured via Vickers hardness testing (type KBW 10-V) with a test force of 49.03 N (HV5) at the same longitudinal cuts as the microstructural analysis. The data, shown in Section 3, consisted of four hardness indentations along the vertical center line of the samples. In addition, uniaxial compression tests were carried out in small cylinders with the of dimensions Ø 5 mm × 7 mm (mechanically machined from built samples with Ø 7 mm × 15 mm) at a compression speed of 10^−3^ s^−1^ using an Instron 4505 equipped with a Hegewald and Peschke control system. For each combination of powder variant and scan velocity the characteristic parameters were determined for four samples. The Young’s modulus was determined via the dynamic resonance frequency method on Ø 5 mm × 50 mm round bars made of AlSi50 p. and AlSi70 Al + Si, which were built with a scan velocity of 1000 mm/s. 

## 3. Results and Discussion

### 3.1. Porosity and Cracks

**Figure 2:**
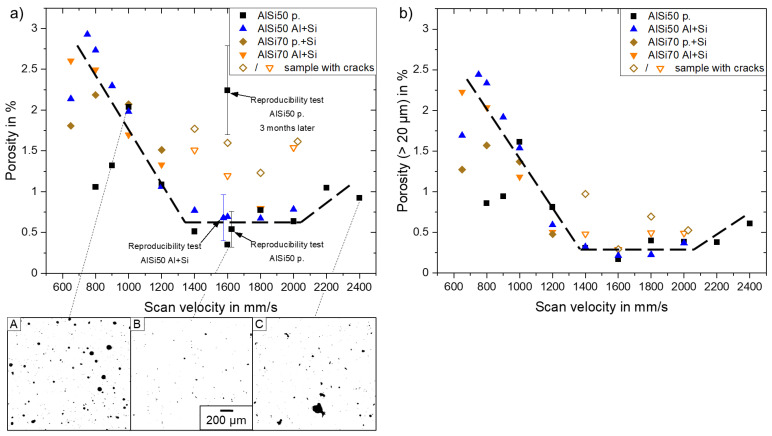
(**a**) Porosity vs. scan velocity for AlSi50 and AlSi70 samples built from different powder types. Binary images of AlSi50 p. samples built with 1000 mm/s (**A**), 1600 mm/s (**B**) and 2400 mm/s (**C**). (**b**) Same as (**a**), but only pores with diameter >20 µm were considered.

In Figure 2a, the measured porosities of all samples are shown in their dependence on scan velocity, powder type and Si content. Herein, one data point represented one sample. The measured values were between 0.4% and ~3% considering all variants. To verify these results, a reproducibility test with eight identical AlSi50 p. samples built with 1600 mm/s within one run was carried out, resulting in an average porosity of 0.54% and a doubled standard deviation of 0.22%. Since the scattering between the samples was much lower than the variations observed in dependence on scan velocity and the different material types, the latter ones could be identified as real effects and not irregularities from the PBF–LB/M process itself. 

The pre-alloyed AlSi50 p., which represents the most conventionally used powder type option, showed its porosity minimum at ~0.5% between 1400 mm/s and 2000 mm/s. In this scan velocity range, only small pores occurred in the polished cuts, as visible in binary image B. One possible cause of these pores could be hydrogen, which is generated through the interaction between the laser beam and residual moisture on the particles’ surfaces [17]. With decreasing scan velocity, the porosity rises due to the formation of bigger keyhole pores resulting from a too high energy input [18]. These pores had a spherical shape, as can be observed in binary image A. Likewise, the porosity also increased at scan velocities ≥2200 mm/s, but the geometry of the occurring pores changed from a spherical to an irregular shape (compare binary image C). These pores, lacked fusion defects, and were formed due to a non-sufficient energy input resulting in incompletely melted zones [19]. A similar dependency of the porosity on the scan velocity was reported by Mueller et al. [12] for pre-alloyed AlSi40 with a minimum porosity of approximately 1% at 1350 mm/s. 

In contrast to the homogeneous powder properties of the AlSi50 p., the elemental Al and Si powders were differentiated in morphology and size, which could lead to inferior part quality when using the blended AlSi50 Al + Si powder. However, the AlSi50 Al + Si showed its porosity minimum at ~0.7% between 1400 mm/s and 2000 mm/s, which was very close to the minimum of the AlSi50 p. powder, and showed increased porosities at slower scan velocities. The porosity determined by the reproducibility test averaged out at 0.68% with a double standard deviation of 0.28%. Consequently, the two different powder approaches can be seen as equivalent in relation to porosity and, so far, the usage of the blended powder resulted in no disadvantage. This was in good agreement with the results of Garrard et al. [13], who showed similar porosities of AlSi40 samples made of pre-alloyed and blended powders.

Furthermore, the measured porosities were in the range of published values for AlSi50, lying between <2% [10] and approximately 0.1% [15]. Nevertheless, the wide scattering within the same material could either be caused by the process parameters or by the porosity measurement itself, because the resolution of the technique used determined the amount of detectable pores Alongside the optical measurement at cuts, nondestructive computer tomography (CT) is a common method used in additive manufacturing, but is rather limited in resolutions up to ~20 µm [20]. To mimic the results which would be obtained from a CT measurement, Figure 2b shows the porosity of the same samples, but considering only pores larger than 20 µm in diameter. It is obvious, that all porosity values decreased. However, while at slower scan velocities the porosities did not change very much due to the presence of large keyhole pores (compare image A in Figure 2), the porosities of both AlSi powder material variants, between 1400 mm/s and 2000 mm/s, decreased to about 0.3%.

The similarity in porosity, regardless of which powder type was used, appeared also in the case of the AlSi70 variants, namely AlSi70 p. + Si and AlSi70 Al + Si. Compared to the AlSi50 samples, the porosity increased from ~0.7% to approximately 1.5% between 1200 mm/s and 2000 mm/s. However, the “simulated” CT measurement showed porosities of the AlSi70 samples of a similar level as the AlSi50 samples. In consequence, the AlSi70 samples, in particular, contained a conspicuous quantity of small pores, which would not be detected in a CT measurement. As the AlSi70 samples were built three months later than the AlSi50 samples, an ageing of the powders and an absorption of moisture was assumed to be a possible explanation for the increase in the number of fine pores. A second reproducibility test with AlSi50 p. powder confirmed this hypothesis. An extreme increase of fine pores <20 µm (approximately 2000%) resulted in a porosity of >2% (see Figure 2a) in this test. Hardness measurements of the old and new samples built within the reproducibility tests showed that the fine pores did not influence the hardness. Probably, these pores were not crucial for the mechanical performance, but maybe for thermo–physical or electrical properties of the alloys. This must be considered during material development when carrying out sample analysis by means of the CT method.

The occurrence of cracks in Al-high Si material is attributed to its low fracture toughness in combination with high thermal stresses induced by steep thermal gradients, high solidification rates and mismatch in thermal expansion between the AlSi material and the base plate (commonly made of Al) used [15]. The influence of process parameters on the thermal gradient and the solidification rate can be described by the energy density E = P/(v∗d∗h) [J/mm^3^], which is a function of laser power P, scanning speed v, hatch distance h and layer thickness d. Cracking in AlSi40 samples was reported for energy densities <50 J/mm^3^ [12] or <66 J/mm^3^ [13], respectively. In the case of AlSi50 material, Hanemann et al. [15] observed cracks for energies ≤111 J/mm^3^, while crack-free samples were obtained by Kang et al. [10] only at even significantly higher energies ≥231 J/mm^3^. Hence, higher energy densities are supposed to lead to reduced thermal gradients and solidification rates and, therefore, should counteract crack formation in Al-high Si alloys. However, all produced AlSi50 samples in this work (energy range was 24–97 J/mm^3^) showed no cracks in volume, independent of the powder source. This contrasted with the earlier findings described above and pointed out that energy density is a not sufficient parameter to explain the cracking behavior of these alloys. Nevertheless, the results showed that production of crack-free AlSi50 samples, with low porosities, was possible, even at lower energy densities. 

In the case of higher Si content, cracks occurred in the AlSi70 samples built with scan velocities of ≥1400 mm/s. They were probably caused by the higher mismatch of thermal expansion with the Al substrate, in combination with the high thermal stresses induced by high scanning velocities. This trend of higher crack sensibility at high scan velocities was also observed for AlSi40 [12,13]. A common counter measure in the literature is a heated baseplate to reduce the thermal stresses [21], but the PBF–LB/M machine used here was not equipped with such a heater system, so this was not further investigated. Nevertheless, it could be successfully demonstrated that crack-free AlSi70 samples with porosities down to 1.25% (1 µm pore resolution) or ~0.5% (CT resolution) could be produced by the PBF–LB/M method using moderate scan velocities ≤1200 mm/s.

### 3.2. Microstructure

**Figure 3:**
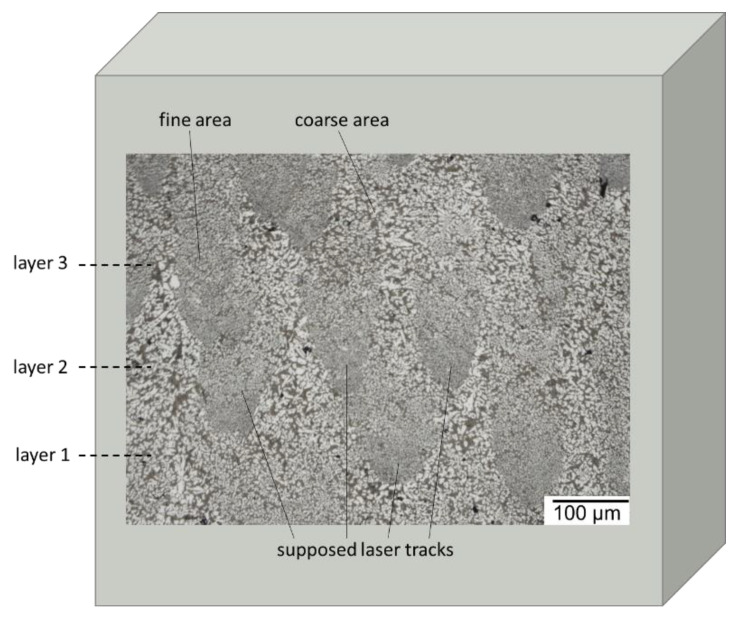
Light microscope image of an etched longitudinal cut of an AlSi50 sample with indicated possible laser tracks and layers.

Resulting from the layer-by-layer buildup, additively manufactured, Al-high Si alloys develop a characteristic microstructure, which consists of two different characteristic areas [12,15,16], as shown in Figure 3. On the one hand, in the center of the melting tracks, fine grains occurred, consisting of primary Si crystals. This region is referred to as the “fine area”. On the other hand, in the overlapping zones and the periphery of the melting tracks coarser primary Si crystals formed, referred to as the “coarse area”. The different grain structure appearance might be correlated to different thermal histories. For example, the temperature distribution and, in consequence, the cooling rate is rather non-uniform inside the melting track [22]. The highest solidification rate occurs in the center of the melt pool, resulting in fine grains. Towards the periphery, the solidification rate continuously decreases, which leads to larger gains. The fine-grained center regions of the melting tracks are typically melted once, while the overlapping coarse-grained regions are melted twice. During the second melting, the formerly built Si phases can partly remain un-melted, so they can act as nucleation sites for new larger Si phases growing during the second solidification [4].

**Figure 4:**
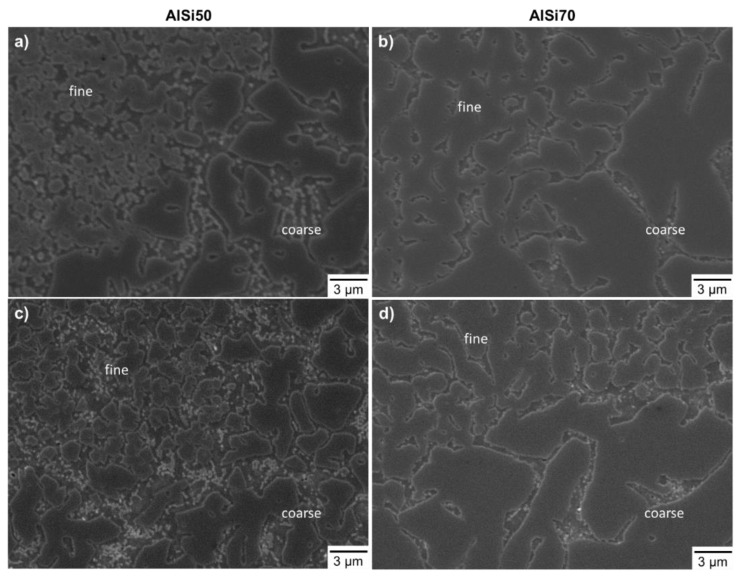
SEM micrographs of the microstructure of: (**a**) AlSi50 p.; (**c**) AlSi50 Al + Si; (**b**) AlSi70 p. + Si and (**d**) AlSi70 Al + Si, recorded in the transition of the fine and coarse area. All samples were built with 1400 mm/s.

Figure 4 shows the microstructure of the transition region between fine- and coarse-grained areas for all considered powder variants. In the fine area of the AlSi50 p. sample, the primary Si particles had a branched or blocky morphology with rounded edges, while they were embedded in a single-phase matrix, which was probably a super saturated solid solution of aluminum [4,16]. The morphology of the primary Si in the coarse area changed into polygonal particles with partly sharp edges, which were surrounded by a eutectic matrix with eutectic Si and an Al solid solution. At the transition between these areas the particles were sometimes connected with each other, which was the biggest difference in comparison to the AlSi50 Al + Si, where the areas were not connected via the particles. Furthermore, the particles’ edges of the AlSi50 Al + Si were more rounded, and the matrix seemed to contain more eutectic Si, which could be related to impurities acting as nuclei for the Si.

Within the AlSi70 samples, the roundly edged primary Si particles built a network in both the fine and the coarse areas. Again, the contiguity in the transition area was more developed in the case of the AlSi70 p. + Si than for that of AlSi70 Al + Si. In analogy to the AlSi50 samples, the interspace of the Si-network was composed of eutectic Si and Al solid solution. 

Thus, it can be stated, that the main differences between the microstructures of the four alloy variants were the strongly developed network of primary Si in the AlSi70 samples, compared to the mostly separated Si-phases in the AlSi50 samples, and the higher contiguity in the samples produced with the pre-alloyed powders, AlSi50 p. and AlSi70 p. + Si. The latter could be caused by different interaction behaviors of the laser beam with the powders, e.g., in relation to different laser absorption ratios [23]. Due to the high reflectivity of the Al powder, less energy could be absorbed by the blended powder beds, which might lead to a narrower melt pool. Consequently, the overlapping of the melting tracks, as well as the contiguity of the Si phases in those regions, could be reduced.

**Figure 5:**
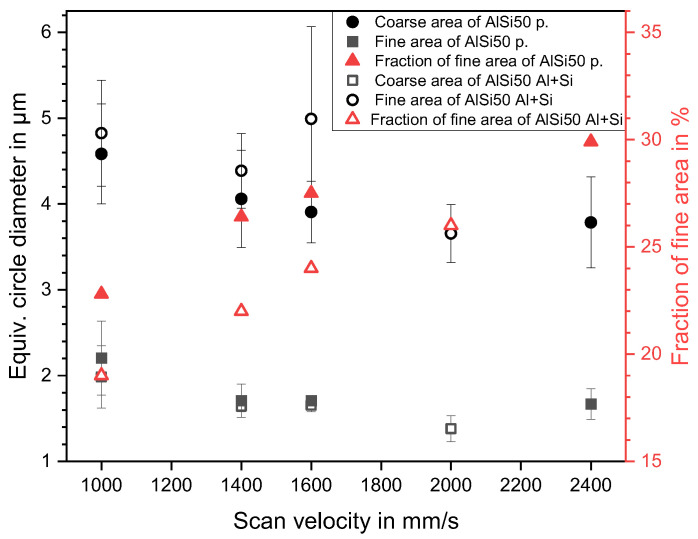
The equivalent circle diameter of the primary silicon and the fraction of the fine area in dependence on the scan velocity for AlSi50 p. and AlSi50 Al + Si samples.

A characteristic parameter to describe the microstructure of AlSi alloys is the size of the primary Si phases formed during the fast cooling of the melt. While the particle size could not be determined in the AlSi70 samples, due to the high contiguity of the Si phases, it was measured in the AlSi50 alloys. The results are shown in Figure 5 in dependence on the scan velocity and the powder type.

With an average diameter <5 µm in the coarse area and <2.5 µm in the fine area the additively manufactured primary Si was significantly smaller, compared to the platelike primary Si in the AlSi50 cast structure (>100 µm [4]), attributable to the high cooling rates in the PBF–LB/M process. In comparison to spray deposited AlSi50 (~13 µm [24]), the size of the primary Si particles could be further reduced. Furthermore, the primary Si sizes in the AlSi50 samples were in the range of the values recorded in the literature for PBF–LB/M, which were between 3 µm [10] and 7 µm [15]. Beyond that, in both areas, the primary Si size decreased with increasing scan velocity, which was probably due to the higher solidification rates at higher scan velocities [22]. In our case, the Si primary particle size showed no strong dependency on the powder variant, which contrasted with the findings by Garrard et al. [12], who reported about 18% larger primary Si particles in AlSi40 samples made of blended powders, compared to ones produced with pre-alloyed powder. The different process parameters used, and/or scanning strategy and/or the powder properties, could provide possible explanations for the discrepancy. 

The fraction of the fine-grained areas, also shown in Figure 5, was 19–26% for the AlSi50 Al + Si and, hence, about 3% lower than for the AlSi50 p. + Si samples (22–30%). Again, this might be correlated to the laser absorption behavior of the different powder beds. The lower energy absorption by the Al + Si powder blend led to a reduced cooling rate inside the melting tracks and, finally, to a slightly coarser grain structure. In both cases, the fraction rose with increased scan velocity, caused by the higher solidification rates. 

Summing up, the primary Si became smaller in all areas, as well as the fraction of the fine areas increasing at higher scan velocities, which correlated to an increased solidification rate. The results of both powder variants were very similar, and only the Al + Si blend resulted in a little lower fraction of fine areas.

### 3.3. Mechanical Properties

#### 3.3.1. Hardness

**Figure 6:**
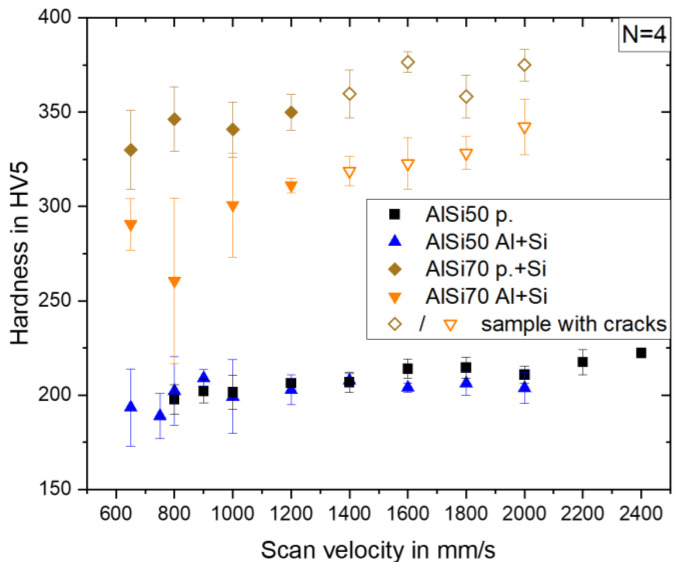
Vickers hardness vs. scan velocity for all AlSi50 and AlSi70 material variants.

The hardness values obtained from all AlSi50 and AlSi70 samples are shown in Figure 6. Firstly, it is obvious, that the hardness generally increased with increasing scan velocity, which was linked to the continuous refinement of the primary Si phase in the microstructure. The two AlSi50 variants exhibited comparable hardness values of ~190 HV5 at low scan velocities. This was in good agreement with the observations by Kang et al. [10], who measured a hardness of 188 HV0.3 in an AlSi50 sample produced with 500 mm/s. 

Even with increasing scan velocity, both variants showed comparable hardness trends ending up with 222 HV5 for AlSi50 p. (at 2400 mm/s) and 204 HV5 for AlSi50 Al + Si (at 2000 m/s). Due to the higher Si content and the denser network of the Si primary phases, the hardness, considering only crack-free samples, and, thereby, a highest scanning velocity of 1200 mm/s, increased to 350 HV5 for AlSi70 p. + Si and 311 HV5 for AlSi70 Al + Si. Here, the samples built from the powder blend containing the pre-alloyed powder showed a significantly higher hardness in comparison to the one produced by the powder blend of Al + Si. This could be attributed to the higher contiguity of the primary Si phases, as shown in Figure 4d. A comparison to a published hardness value of a spray deposited AlSi70 alloy, which was significantly lower at 270 HV5 [7], underlines the attractiveness of the additive manufacturing approach for the production of these alloys. 

#### 3.3.2. Compressive Strength and Ductility

**Figure 7:**
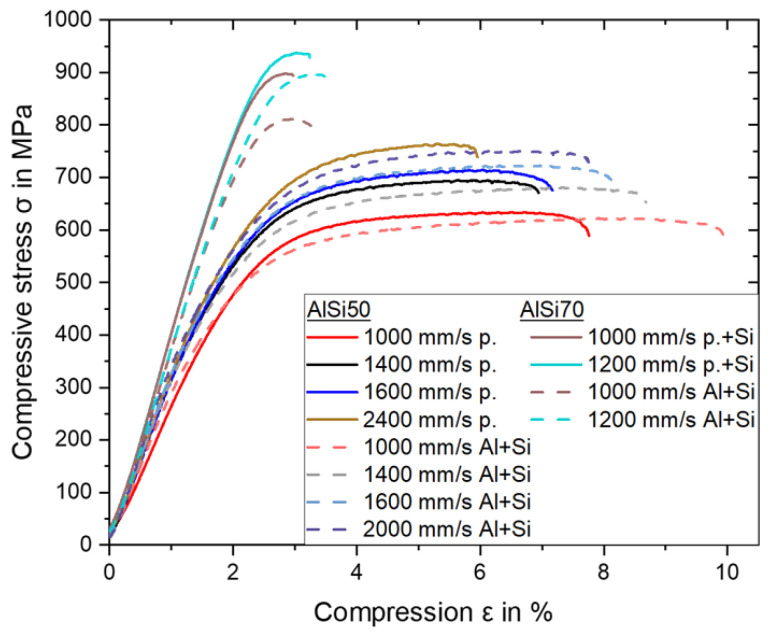
Compressive stress-compression diagram for samples of both AlSi50 variants fabricated with four scan velocities and for samples of both AlS70 variants produced with two scan velocities.

The course of the compressive strength–compression curves in Figure 7 can be divided into three parts. Firstly, the material deforms were merely elastic, so the compressive stress rose in a straight line. When reaching 0.2% compressive offset yield strength (0.2% OYS), the material started to deform plastically, e.g., at 450 MPa for the presented AlSi50 p. 1000 mm/s graph. In this section, the curve flattened, and the round bar started to bulge with advancing compression; but the compressive stress was still rising. In the last section, the compressive stress decreased, and the sample failed and sheared at a 45° angle to the compression direction. At this point, the compressive strength (CS) and fracture compression were reached, which were 590 MPa and 5.2% for the AlSi50 p. 1000 mm/s curve shown.

Analogous to the hardness, the 0.2% OYS and the CS also rose with increasing scan velocity, independent of the Si content and powder variant. Simultaneously, the fracture compression decreased. In the case of AlSi50 p., a rise in scan velocity from 1000 mm/s to 2400 mm/s caused an increment of the 0.2% OYS from 459 ± 27 MPa to 576 ± 35 MPa and a rise of CS from 584 ± 15 MPa to 742 ± 11 MPa, while the fracture compression declined from 5.6 ± 0.5% to 3.7 ± 0.3%. 

With increasing scan velocity, finer grain structures formed, which increased the strength according to the Hall–Patch mechanism [25,26]. Moreover, the above-mentioned trends of the decreasing primary Si size and the higher fraction of fine area with increasing scan velocity could enhance this effect. Conditioned by the higher fraction of the fine area a decrease of the extensive matrix zones in the coarse areas occurred. As the matrix was more ductile than the primary Si, the reduced matrix areas resulted in lower ductility and fracture compression in the materials built with higher scan velocities. Furthermore, the matrix–particle interface enlarged due to the refinement of the primary Si particles. This hindered the dislocation movement, which also led to reduced ductility. 

The obtained values were in good agreement with the findings by Jia et al. [4], who measured a CS of ~667 MPa and a fracture compression of ~4% in a sample built with a scan velocity of 1455 mm/s. The value for the 0.2% OYS of approximately 600 MPa was notably higher than the measured value of 475 ± 30 MPa at 1400 mm/s in this work. Since all process parameters used were quite comparable, one reason for the discrepancy could be the sample geometry. Jia et al. [4] used round bars with dimensions of Ø 6 mm × 25 mm, which corresponded to a height–diameter ratio >4 (in contrast to <2 used in this work). At this dimension, a buckling or bending of the samples cannot be ruled out, according to the DIN 50106:2016-11 [27], which could lead to a more inaccurate measurement.

Compared to the AlSi50 p. samples, the AlSi50 Al + Si samples showed lower 0.2% OYS, but similar CS. On the one hand, the lower contiguity of the primary Si led to a more ineffective load redistribution by the particles, which could cause higher stresses in the matrix of the AlSi50 Al + Si samples at the same external forces and activate dislocation sources [28]. This could be one reason for the inferior 0.2% OYS of the AlSi50 Al + Si samples. On the other hand, the larger matrix–particle interface could create a bigger backlog effect for the dislocations, leading to almost identical compressive strengths [29,30]. Furthermore, the AlSi50 Al + Si samples exhibited about 2% higher fracture compressions than the AlSi50 p. samples with a maximum of 7.3 ± 0.8% at 1000 mm/s. This could possibly be attributed to differences in the microstructure, like the particle morphology and the contiguity in the transition areas. In the AlSi50 p. samples, the primary Si particles were more often edgy, which could induce stress peaks decreasing ductility [12]. Additionally, the matrix size between the fine and coarse areas, which was larger in the transition area of the AlSi50 Al + Si samples, due to the less connected primary Si phases, could partly compensate the stress by plastic deformation. 

In the same way as for the hardness, the CS also rose with increasing Si content. At the same scan velocity of 1000 mm/s the CS rose from 584 ± 15 MPa for AlSi50 p. and 595 ± 17 MPa for AlSi50 Al + Si to 897 ± 6 MPa for AlSi70 p. + Si and 805 ± 19 MPa for AlSi70 Al + Si. The strength increment was accompanied by a decrement in ductility with the result that the fracture compression reduced to 0.7 ± 0.1% for AlSi70 p. + Si and 1.2 ± 0.1% for AlSi70 Al + Si. The increment of the scan velocity up to 1200 mm/s, which was the highest velocity resulting in crack-free samples, resulted in no difference in the fracture compression, probably based on the small enhancement, or on the already low level of ductility. Apart from that, the scan velocity showed an influence on the strength, such that the CS increased to 935 ± 5 MPa for AlSi70 p. + Si and 870 ± 27 MPa for AlSi70 Al + Si with an increment from 1000 mm/s to 1200 mm/s.

The significant enhancement in strength from the AlSi50 to the AlSi70 samples could be attributed to the higher contiguity of the primary Si, which enabled more effective load redistribution on this skeletal structure and a shielding of the matrix [29,31]. As the contiguity was higher in the AlSi70 p. + Si samples, they showed superior strengths compared to the AlSi70 Al + Si samples. Otherwise, the dominant skeletal structure was extremely brittle resulting in the decrement of ductility compared to AlSi50, in which the primary Si phase was embedded in a more ductile Al and Si matrix.

To sum up the trends during compressive testing, strength was enhanced with increasing scan velocity and Si content but with a simultaneous reduction in ductility. In addition, the use of the blended powder could improve the ductility of Al-high Si alloys without (AlSi50), or with only small (AlSi70) loss of strength.

#### 3.3.3. Young’s Modulus

**Figure 8:**
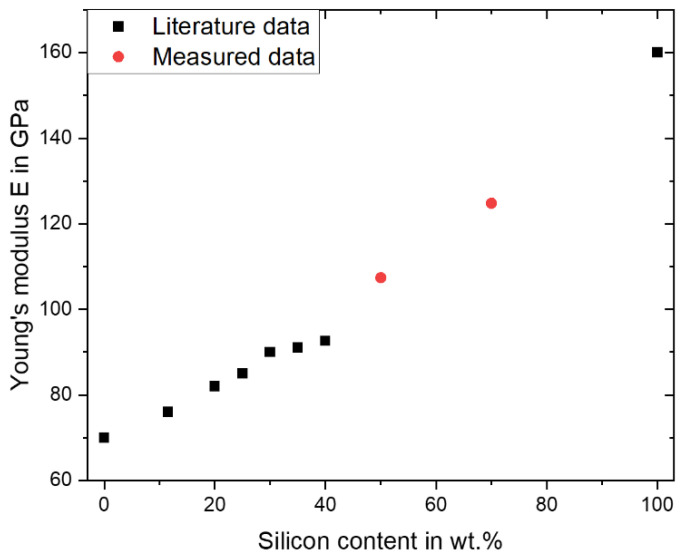
Literature data and measured data (AlSi50 p. and AlSi70 Al + Si from this work) for the Young’s modulus depending on the silicon content. The literature data was for pure aluminum and silicon [24], AlSi10Mg [11], AlSi20, AlSi25, AlSi30, AlSi35 all from [32] and AlSi40 [12].

Due to the higher Young’s modulus of Si, of approximately 160 GPa, compared to Al at about 70 GPa, the Young’s modulus in Al-high Si alloys could be increased by increasing the Si content. This is illustrated in Figure 8 and by literature data on AlSi alloys, up to 40 wt.% Si. In addition, the measurement results of one AlSi50 p. sample and one AlSi70 Al + Si sample (both built with 1000 mm/s) of this work are shown. The Young’s modulus increased from 93 GPa for AlSi40 [12] to 105 GPa for the AlSi50 p. sample, and further increased up to 124 GPa in the case of the AlSi70 Al + Si sample, which continued the trend evident in the literature data. Despite the fact that the trend did not proceed in an exact linear correlation, according to the rule of mixture, but in a curve below the straight line, it was possible to adjust, and also to further increase, the Young’s modulus via accommodation of the Si content.

## 4. Conclusions

In this work, a fundamental study on the additive manufacturing of Al-high Si alloys (AlSi50 and AlSi70) by means of PBF–LB/M technology was carried out. It was demonstrated, for the first time, that it is possible to produce crack-free and highly dense samples, even up to an extremely high Si content of 70 wt.%, which was realized by adding elemental Si to pre-alloyed AlSi50 powder, or by directly mixing Al and Si. 

The porosity of the samples strongly depended on the scan velocity and was influenced only in a minor way by the powder source used. However, ageing of the powders could lead to massive insertion of small pores of <20 µm in diameter, which were only visible by microscopic analysis. This should be considered during alloy development with respect to pore affected properties.

The mechanical properties of the Al-high Si alloys were clearly correlated to the microstructure. Higher scan velocities led to a decrease in grain size and, consequently, to an increase in hardness, compression strength and Young’s Modulus. A significant improvement could be achieved by increasing the Si content from 50 to 70 wt.%, inducing the formation of a dense network of primary Si phases. On the other hand, some loss of ductility existed, which could be partly prohibited using elemental powder blends. 

Presumably the cracking issue, which sporadically appeared in the AlSi70 samples at higher scan velocities, is the main issue for establishing AM of Al-high Si alloys with extremely high Si content. Hence, further development of adequate countermeasures, especially in the course of upscaling the sample size, are required.

## Data Availability

Not applicable.
